# Prediagnostic prescription patterns in multiple sclerosis: a UK-based longitudinal linked data study

**DOI:** 10.3399/BJGP.2025.0210

**Published:** 2026-04-21

**Authors:** Baoyue Zhang, Oleg Blyuss, Kirsten D Arendse, Luke TA Mounce, Ruth Dobson, Garth Funston

**Affiliations:** 1 Centre for Cancer Screening, Prevention and Early Diagnosis, Wolfson Institute of Population Health, Queen Mary University of London, London, UK; 2 Department of Pediatrics and Pediatric Infectious Diseases, Sechenov University, Moscow, Russia; 3 Institute of Health Research, University of Exeter, Exeter, UK; 4 Department of Neurology, Royal London Hospital, Barts Health NHS Trust, London, UK

**Keywords:** erectile dysfunction, gastrointestinal symptoms, migraine, multiple sclerosis, primary health care, urinary tract infection

## Abstract

**Background:**

Early detection of multiple sclerosis (MS) could improve patient outcomes. Changes in clinical activity before MS diagnosis may represent ‘diagnostic windows’ for earlier detection.

**Aim:**

To determine whether certain prescriptions are more common in patients with MS and examine prescription patterns prediagnosis in the UK.

**Design and setting:**

Case–control study using routinely collected primary care data. Patients in the case group had an MS diagnosis (2001–2022) and patients in the control group were matched by birth year.

**Method:**

Multivariable conditional logistic regression adjusting for sex and socioeconomic status (SES) examined associations between MS diagnosis and prescriptions for anxiety/depression, migraine, lower urinary tract symptoms (LUTS), urinary tract infection (UTI) treatments, sleep disorder, gastrointestinal (GI) symptoms, and erectile dysfunction (ED) in the 0–2, 2–5, 5–10 years prediagnosis/match date. Poisson regression adjusting for age, sex, and SES estimated the inflection points in the case group at which the rate of prescriptions changed from baseline, identifying the start of diagnostic windows.

**Results:**

In total, there were 9662 patients in the case group and 56 455 in the control group. Positive associations were found between seven prescriptions and MS diagnosis, with highest odds ratios (3–7 times) in the 2 years prediagnosis. In the all-prescription analysis, inflection points were identified for: GI (69–72 months, 95% confidence interval [CI] = 51 to 90), LUTS (69–72 months, 95% CI = 30 to 111), UTI (66–69 months, 95% CI = 45 to 90), migraine (63–66 months, 95% CI = 27 to 102), ED (60–63 months, 95% CI = 30 to 93), and anxiety/depression (9–12 months, 95% CI = 3 to 18).

**Conclusion:**

Prescriptions for symptoms of possible MS were more common in the case group than the control group up to 10 years prediagnosis. Prescription rates changed years prediagnosis, indicating potential opportunities for earlier detection, but broad CIs demonstrated uncertainty regarding diagnostic window duration.

## How this fits in

Previous research indicates that patients with multiple sclerosis (MS) may experience a prodrome with more frequent healthcare usage (including hospital admissions, physician and hospital encounters, and prescriptions) in the 5–10 years before a confirmed diagnosis of MS. Changes in clinical activity before MS diagnosis may represent ‘diagnostic windows’ in which earlier detection may be achieved for some patients. This study found that prescriptions related to anxiety/depression, urinary tract infection treatments, migraine, gastrointestinal symptoms, lower urinary tract symptoms, sleep disorder, and erectile dysfunction were more common in the case group than the control group up to 10 years prediagnosis, and that rates of several prescriptions changed from baseline rates in patients with MS up to 6 years prediagnosis. These findings indicate that there may be potential prolonged diagnostic windows for patients with some MS-related symptoms, in which earlier diagnosis is possible.

## Introduction

Over 2.8 million people worldwide, and 150 000 people in the UK, have multiple sclerosis (MS).^1–3^ MS is an archetypal complex disease, with both genetic and environmental risk factors implicated, including Epstein–Barr virus infection, low vitamin D levels, and adolescent obesity.^4,5^ It is an incurable, progressive disease that leads to irreversible neurological damage and increasing disability over time.^6,7^ Early detection of MS is important to optimise treatment with disease-modifying therapies, which can protect brain or spinal cord function, thereby preventing further disease-related decline and worse outcomes.^4,7,8^

‘Diagnostic windows’ refer to timeframes before diagnosis when frequency of clinical events, such as symptom reporting, medication prescriptions, and abnormal test results, in patients in whom the condition is as-yet-undiagnosed changes detectably from the healthy population/baseline rate.^9–11^ For some patients, different clinical events and changes can occur at different timepoints before diagnosis, offering opportunities for earlier detection.^9–11^ Recently, mounting evidence indicates that patients with MS show elevated serum neurofilament light chain,^12^ white matter lesions,^13^ prodromal symptoms,^14–18^ and more frequent healthcare usage (including hospital encounters and prescriptions)^17,19–23^ 5–10 years before a confirmed diagnosis of MS.

Although accumulated evidence demonstrates the potential diagnostic windows of MS, their specific length, features, and timing remain unclear. Prescriptions may serve either as indicators of underlying disease risk or as proxies for early symptoms. Using prescription data, which are well recorded in GP records, to identify diagnostic windows has been widely utilised in cancer research, but remains unexplored for MS in the UK, to the authors’ knowledge.^10,11^ Most previous studies used analysis methods to examine the crude prodromal phase, without identifying the key deviation timing and explicit diagnostic window length.^23^

In this study, the aim was to determine whether seven types of prescriptions, which may be used to treat MS-associated symptoms, are more common in patients with MS in the 10 years prediagnosis compared with a control group. Then, longitudinal data before MS diagnosis were used to identify ‘inflection points’ (the point at which the prescription rate deviates from the baseline trend) to estimate the length of diagnostic windows where earlier diagnosis may be achieved for certain groups.

## Method

### Study design and population

The Clinical Practice Research Datalink (CPRD) Aurum contains routinely collected clinical records from primary care, with linkage to other national datasets.^24^ In this study, data from a previously conducted CPRD study was used,^25^ which comprised CPRD Aurum and small-area-level data, to perform a population-based retrospective case–control study and a case-only study.

A research team that included neurologists developed a broad diagnostic code list for MS in CPRD for initial ‘case’ identification.^25^ All codes in the list were subsequently classified into three levels of diagnostic confidence: definite, probable, and possible.^25^ Codes highly specific for MS were categorised as definite diagnostic codes, whereas codes indicative of other inflammatory or demyelinating conditions were classified as probable or possible MS diagnostic codes.^25^ These confidence levels were then used to guide the subsequent exclusion process.

Patients who were potential case patients (with ≥1 MS diagnostic code) and those who were potential control patients (with no MS code) were initially identified from CPRD Aurum, with those in the control group matched to the case group at a 1:10 ratio by year of birth. Each case patient had an index date, defined as the date of the first recorded MS diagnostic code during the study timeframe. Each control patient was assigned the same index date as their matched case patient. The following selection criteria were used (detailed in Supplementary Boxes S1 and S2).

For the case group:

- ≥5 years of prior GP registration at the index date;
- aged ≥18 years at the index date;
- to improve accuracy of selection, in the current study, the authors applied a more stringent definition than a single-code definition, requiring ≥2 definite diagnostic codes in CPRD Aurum, following the approach used by Jacobs *et al*;^25^ and
- to ensure a homogeneous population, only patients with an index date between 2001 (the first McDonald MS criteria published) and 2022 were included.^26^

For the control group:

- aged ≥18 years at the index date;
- included only if a matched case patient remained eligible after application of the above case criteria; and
- ≥5 years of prior GP registration at the index date.

### Prescriptions of interest

Based on previous evidence of MS prodrome and the clinical consensus of a GP and neurologist, seven categories of relevant prescriptions were selected and defined according to the *British National Formulary* (see Supplementary Box S3). The corresponding prescription code lists are provided in Supplementary Table S1:

- antidepressants/anxiety medications;
- migraine/cluster headache-specific medications;
- lower urinary tract symptoms (LUTS)-related medications (irritative, obstructive, and incontinence);
- urinary tract infection (UTI) treatments-related medications;
- medications for sleep disorder;
- gastrointestinal (GI) medications (including laxatives/antidiarrhoeals/GI antispasmodics and irritable bowel syndrome treatments); and
- medications for erectile dysfunction (ED).

These categories represent prescriptions related to MS symptoms or conditions that may mimic MS.

### Contributing features and confounders

Age, sex, and socioeconomic status (SES) are implicated in both MS^27–29^ and the above medication usage,^30–33^ so were considered as confounders in all analyses. Age was not included in conditional logistic models, as it was the matching variable. Age was derived from birth year with estimated day as 1 January, and was modelled as a categorical variable to allow an exploration of the relationship and to account for step changes. For descriptive analyses, age at the index date was categorised into 10-year groups. In Poisson models, age was time varying and calculated at each period’s midpoint (5-year groups). Index of Multiple Deprivation (IMD) is commonly used for estimating the deprivation level.^34^ It was extracted from linked patient-level small-area-level data, and was classified into five quintiles, with one being the lowest quintile of deprivation and five the highest quintile of deprivation.^34^

### Statistical methods

Descriptive analyses were performed summarising:

- demographic characteristics and the proportion of participants who received a prescription within each drug category during the 10 years before the index date in the case and control groups; and
- the proportion of participants with a prescription in a given category by age, sex, and IMD for the case and control groups.

Statistical analyses were first performed using all prescription records in a given category in the study timeframe, then repeated for the first-ever CPRD prescription record in a given category in the 10 years before the index date, as the initiation of a new prescription type may indicate the emergence of a new symptom. An additional sensitivity analysis was conducted by repeating all analyses in those in the case and control groups with ≥10 years of registration before the index date, to assess the potential impact of including participants who were required to have only 5 years of registration before the index date but were treated as having 10 years of look-back in the main analysis.

Using matched case and control groups, multivariable conditional logistic regression models were applied to examine associations between MS diagnosis and the seven prescription categories in 0–2, 2–5, and 5–10 years pre-index date, adjusting for IMD and sex, calculating odds ratios (ORs) with 95% confidence intervals (CIs).

Multilevel Poisson regression was used to identify inflection points (when the population-level prescription rates changed from baseline) in patients with MS in the 10 years before diagnosis. The study timeframe was divided into 40 equal 3-month intervals (excluding the closest 3 months). Models adjusting for time-varying age, IMD, and sex were run for each potential inflection period, with variables included to address the background rate and ‘inflection period’ variables to capture the change in rate from the background rate.^35–37^ The model with the largest log likelihood was considered the best-fitting model and used to determine the period containing inflection points.^36^ Bootstrapping with 50 iterations was performed to estimate the 95% CIs based on the normal approximation method.^36^ An inflection point was regarded as practically meaningful if its 95% CI lay entirely within the study window (period 1–40).^35,36^ Poisson results were reported in periods. To aid clinical interpretation, the period was also converted into 3-month intervals before the diagnosis date. For a conservative conversion, non-integer 95% CIs were adjusted to 3-month intervals by rounding the lower bound down and upper bound up to the nearest 3-month period. No control group was used, as this approach seeks to identify changes from baseline in the patient group of interest.

Analyses were performed, and graphs and tables generated using Stata (version 18) and R (version 4.2.2).

## Results

### Descriptive results

A total of 66 117 individuals were included in the study (Figure 1). There were 56 455 (85.39%) control patients and 9662 (14.61%) case patients. In total, 2 417 070 related prescription records were identified.

**Figure 1.**
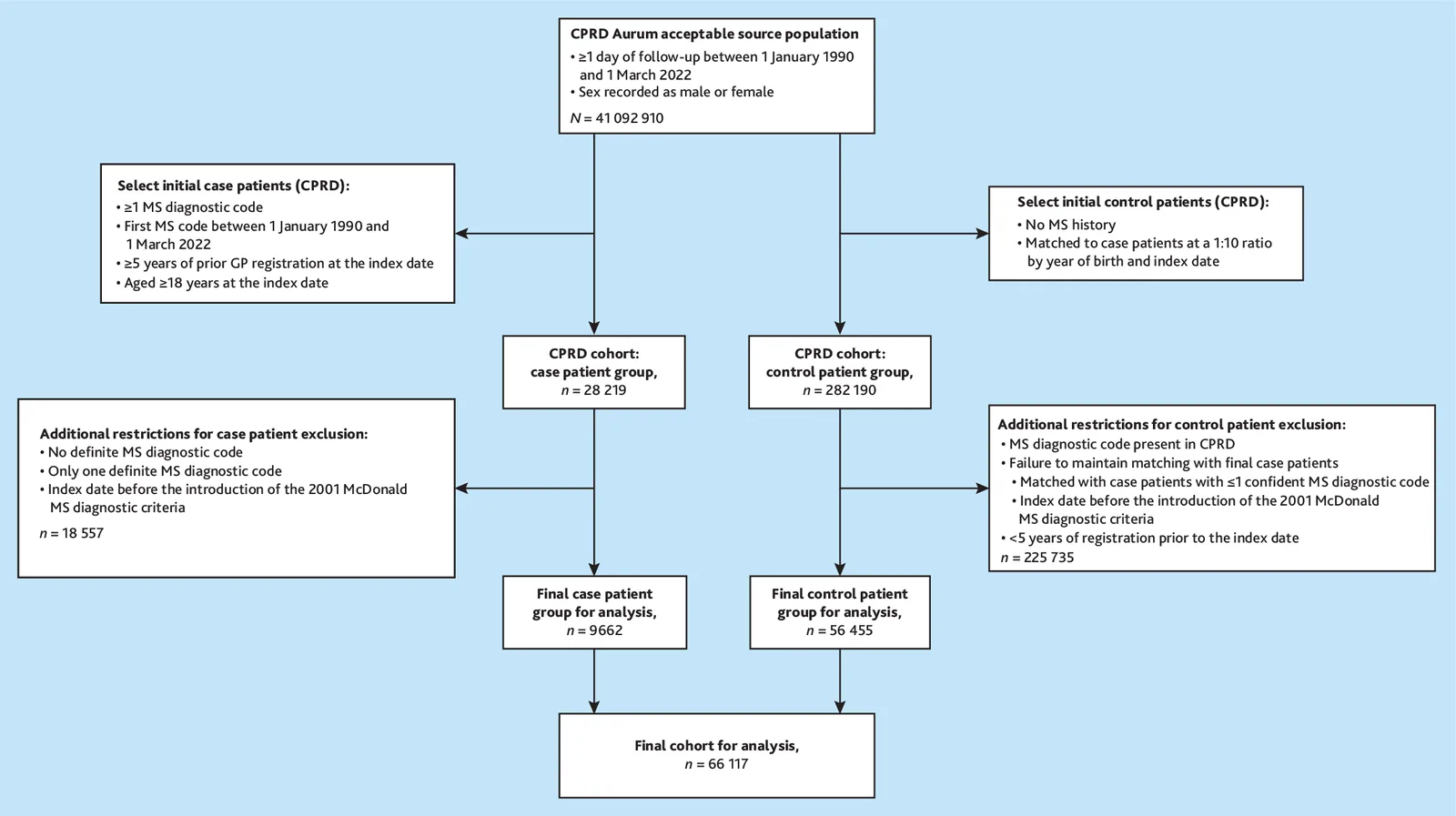
Selection process for the case and control patients. CPRD = Clinical Practice Research Datalink. MS = multiple sclerosis.

Table 1 shows basic demographic information for different groups.

**Table 1.** Demographic characteristics in the case and control groups

	All participants		Case group		Control group	
Characteristic	*n*	%	*n*	%	*n*	%
**Total participants**	66 117	100	9662	14.61	56 455	85.39
**Age at index date, years**						
<30	6573	9.94	1055	10.92	5518	9.77
30–39	15 070	22.79	2138	22.13	12 932	22.91
40–49	23 783	35.97	3085	31.93	20 698	36.66
50–59	14 415	21.80	2227	23.05	12 188	21.59
60–69	4703	7.11	887	9.18	3816	6.76
≥70	1573	2.38	270	2.79	1303	2.31
**Sex**						
Male	30 885	46.71	2836	29.35	28 049	49.68
Female	35 232	53.29	6826	70.65	28 406	50.32
**IMD quintile**						
1 (lowest deprivation)	13 230	20.01	2171	22.47	11 059	19.59
2	12 981	19.63	2111	21.85	10 870	19.25
3	12 556	18.99	1906	19.73	10 650	18.86
4	12 805	19.37	1740	18.01	11 065	19.60
5 (highest deprivation)	11 254	17.02	1500	15.52	9754	17.28
Missing	3291	4.98	234	2.42	3057	5.41

IMD = Index of Multiple Deprivation.

Stratified analysis (see Supplementary Tables S2 and S3) revealed similar patterns for prescriptions overall and first prescription. The case group consistently had a higher proportion of participants with a prescription than the control group, regardless of stratification by age, sex, and IMD quintile. Prescription rates were generally higher in older patients, females (except for LUTS), and the IMD quintile 5 group.

In the 10 years before the index date, the prescription proportion (all prescriptions) of each category increased steadily as the index date approached. The increasing trend was steeper in the case group but gentler in the control group. For first prescription, rates for the case group fluctuated but generally increased over time, whereas the rates for the control group exhibited only minor variations. First UTI prescription showed a distinct pattern, with prescription rate initially increasing and then declining (Figure 2).

**Figure 2.**
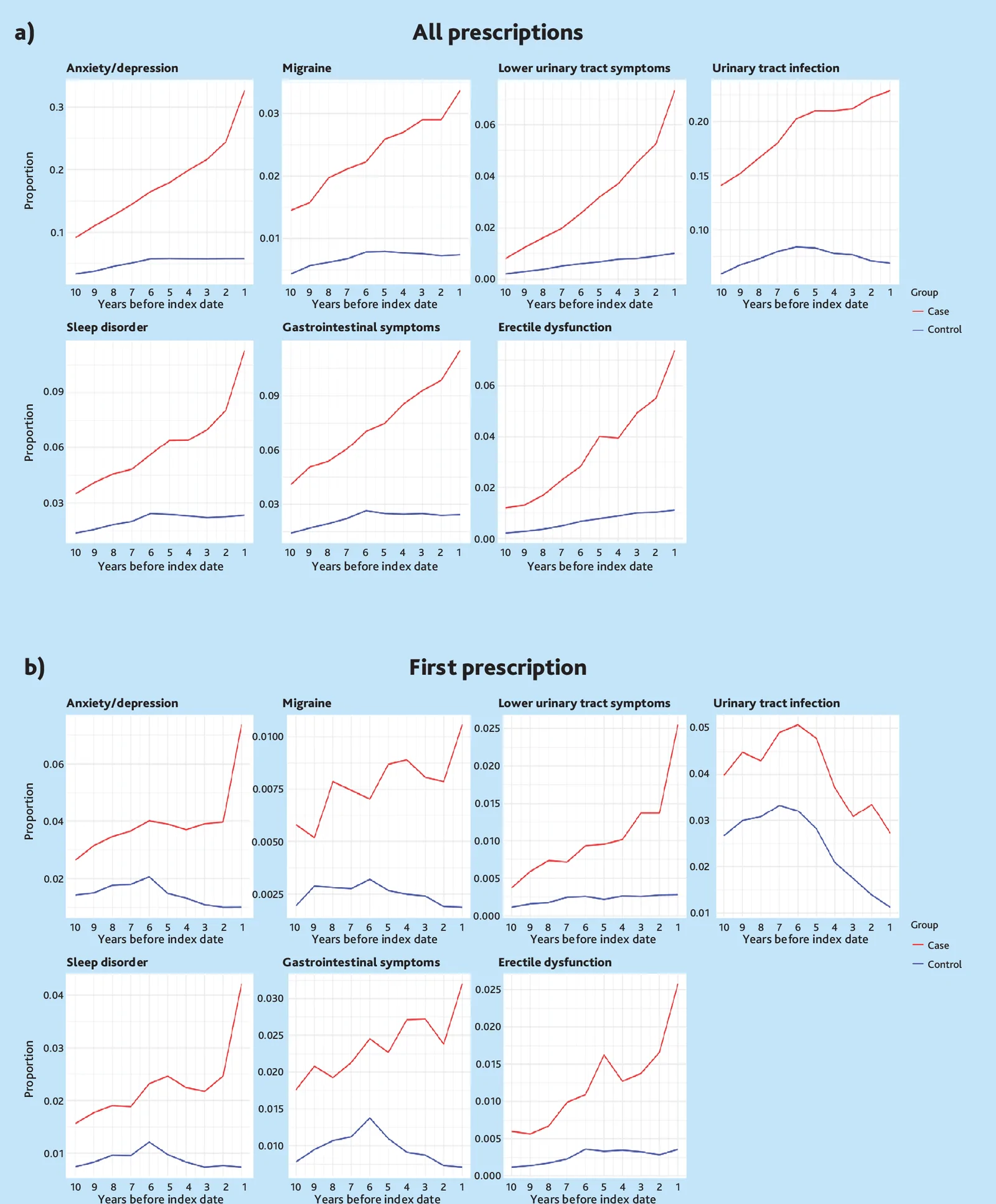
Proportions of participants with a prescription in the 10 years before the index date for the case and control groups: a) all-prescription analysis; b) first-prescription analysis. Years before index date: the lowest value is the year closest to the index date.

### Multivariable conditional logistic regression

Table 2 lists the adjusted results for seven prescription categories in all-prescription analysis. The complete results for multivariable models (including parameters for confounders) are listed in Supplementary Table S4.

**Table 2.** Results of multivariable conditional logistic regression models for seven prescriptions (all prescriptions).^a^

Prescription	Years before the index date					
	0–2		2–5		5–10	
	OR (95% CI)	*P*-value	OR (95% CI)	*P*-value	OR (95% CI)	*P*-value
Anti-anxiety/depression	6.68 (6.31 to 7.08)	<0.001	3.69 (3.49 to 3.91)	<0.001	2.51 (2.38 to 2.66)	<0.001
Migraine	3.51 (3.06 to 4.02)	<0.001	2.70 (2.39 to 3.07)	<0.001	2.15 (1.90 to 2.43)	<0.001
Lower urinary tract symptoms related	7.47 (6.64 to 8.41)	<0.001	4.71 (4.15 to 5.34)	<0.001	3.42 (2.96 to 3.95)	<0.001
Urinary tract infections treatments related	3.71 (3.52 to 3.92)	<0.001	3.08 (2.93 to 3.23)	<0.001	2.49 (2.38 to 2.62)	<0.001
Sleeping disorder	4.37 (4.04 to 4.72)	<0.001	2.75 (2.55 to 2.97)	<0.001	2.22 (2.06 to 2.39)	<0.001
Gastrointestinal	4.21 (3.90 to 4.55)	<0.001	3.01 (2.80 to 3.23)	<0.001	2.17 (2.03 to 2.33)	<0.001
Erectile dysfunction	6.38 (4.99 to 8.15)	<0.001	3.69 (2.89 to 4.72)	<0.001	2.79 (2.10 to 3.69)	<0.001

^a^The lowest value is the year closest to the index date. IMD = Index of Multiple Deprivation. OR = odds ratio

When considering all prescriptions, positive associations were found between each of the seven prescription categories and subsequent MS diagnosis at 0–2, 2–5, and 5–10 years before the index date after adjusting for sex and IMD. Each category had the highest OR in the 0–2 years before the index date. The association was greatest between MS and LUTS-related prescriptions. When considering first prescription, positive associations remained for the seven prescription categories, but ORs were lower for the categories, except for migraine and ED in the 0–2 and 2–5 years time windows (see Supplementary Tables S5 and S6).

### Inflection point: multilevel Poisson regression

#### All prescriptions

Significant inflection points were observed in six prescription categories (Figure 3). GI and LUTS prescriptions changed first, with inflection points at period 24 (95% CI = 18.46 to 29.54 and 95% CI = 11.04 to 36.96), followed by UTI (period 23, 95% CI = 16.55 to 29.45), migraine (period 22, 95% CI = 10.38 to 33.62), ED (period 21, 95% CI = 11.62 to 30.38), and anxiety/depression (period 4, 95% CI = 2.57 to 5.43). The rate of increase in migraine and UTI prescriptions slowed after the inflection point, whereas GI, LUTS, ED, and depression/anxiety rates accelerated. No significant inflection point was found for sleep disorder (period 3, 95% CI = −2.92 to 8.92). For prescriptions other than anxiety/depression, the 95% CIs were broad (spanning 3–7 years), indicating high uncertainty in the inflection point estimation.

**Figure 3.**
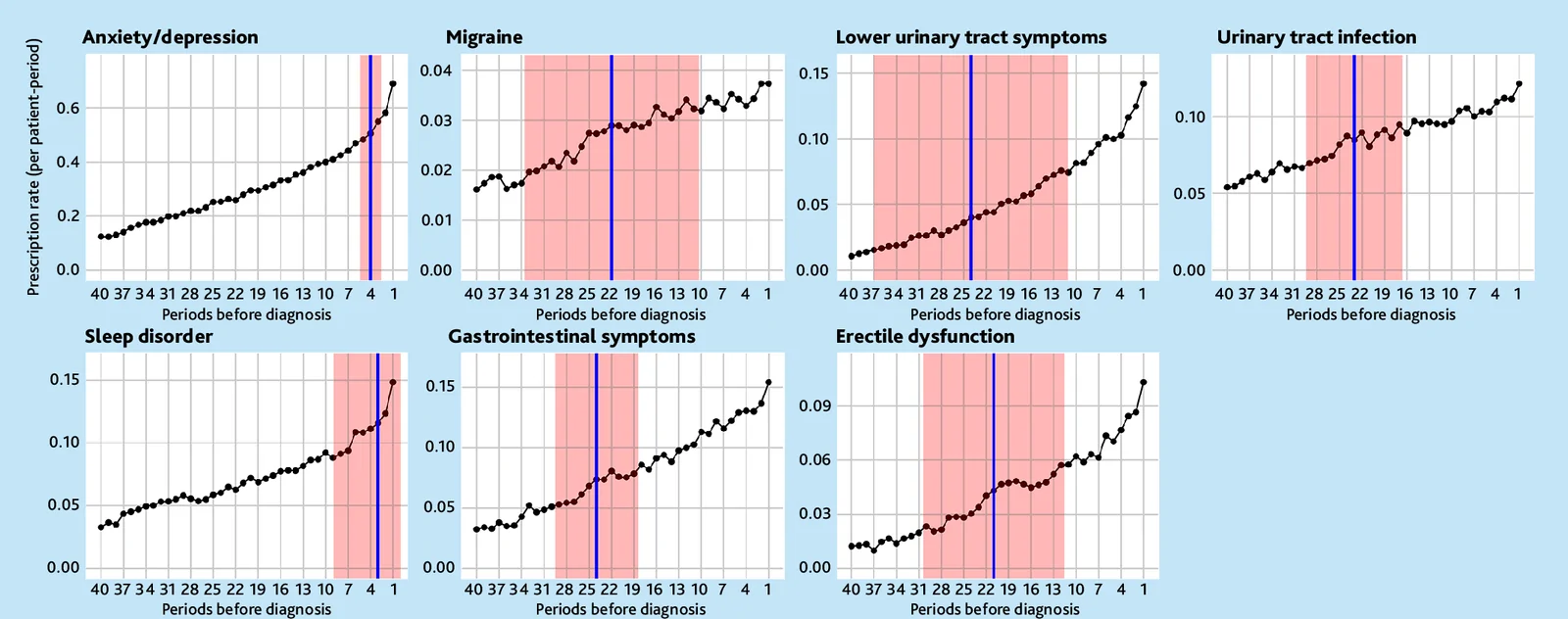
Rates of recordings, inflection points, and 95% CI for inflection points of each prescription category in the 10 years leading up to diagnosis (all prescriptions). Inflection point is indicated by a solid vertical line, shaded area indicates 95% CI. Each period covers 3 months. The lowest value is the period closest to diagnosis.

#### First prescription

Significant inflection points were found for four categories (Figure 4), excluding migraine (period 4, 95% CI = −16.39 to 24.39), GI (period 2, 95% CI = −13.61 to 17.61), and ED (period 2, 95% CI = −20.65 to 24.65). UTI prescriptions changed first at period 22 (95% CI = 19.78 to 24.22) with a decreasing rate. This was followed by anxiety/depression (period 3, 95% CI = 2.09 to 3.91), LUTS (period 3, 95% CI = 1.37 to 4.63), and sleep disorder (period 3, 95% CI = 2.24 to 3.76), with all showing steeper increase rates post-inflection.

**Figure 4.**
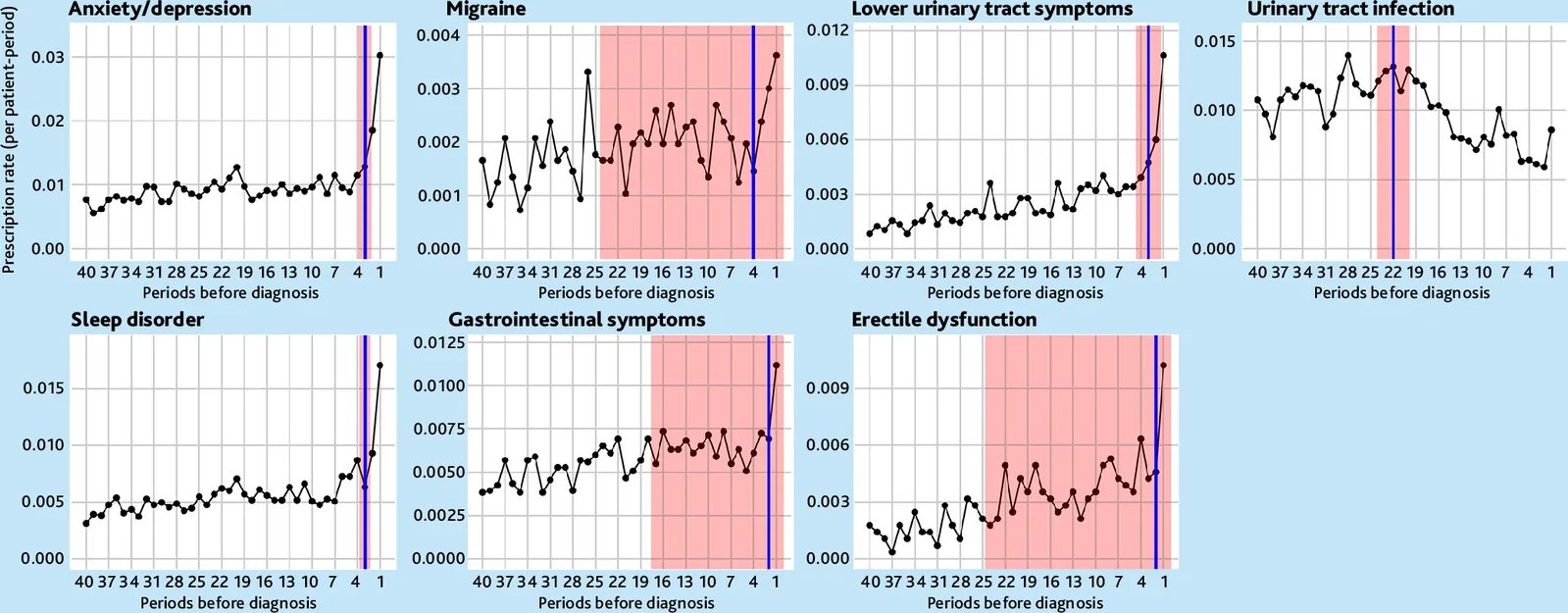
Rates of recordings, inflection points, and 95% CI for inflection points of each prescription category in the 10 years leading up to diagnosis (first prescription). Inflection point is indicated by a solid vertical line, shaded area indicates 95% CI. Each period covers 3 months. The lowest value is the period closest to diagnosis.

The converted month-based results are listed in Supplementary Information S1.

### Sensitivity analysis: 10-year registration cohort

A total of 42 765 participants were included in the sensitivity analysis.

#### Conditional logistic regression

The associations between each of the seven prescription categories and subsequent MS diagnosis remained significant across time windows (0–2, 2–5, and 5–10 years), with the highest ORs observed in the 0–2 years before the index date.

#### Poisson regression

Significant inflection points were observed for depression/anxiety and ED in all-prescription analyses.

Full results are listed in Supplementary Tables S7–S13 and Supplementary Figures S1–S3.

## Discussion

### Summary

Using a case–control design, this study found that seven prespecified categories of prescriptions showed significant associations with subsequent MS diagnosis, with the highest ORs noted for all categories in the 2 years prediagnosis. In the case group, inflection points, at which the rate of prescriptions changed from baseline, were identified for GI, LUTS, UTI, migraine, anxiety/depression, and ED prescriptions between 9 and 72 months before diagnosis. This indicates that the diagnostic window may extend to 6 years prediagnosis for patients with MS who have specific prodromal symptoms/prescriptions, but given very broad CIs, there is uncertainty as to when diagnostic windows start.

### Strengths and limitations

This study used a large routinely collected representative electronic healthcare record data source. To the authors’ knowledge, this is the first study to explicitly measure diagnostic windows for MS.^38^ An important strength is that prescription data are automatically recorded in GP systems rather than being manually coded (like symptoms) resulting in high completeness. A further strength is the use of two statistical approaches: a case–control design using conditional logistic regression, which enabled assessment of associations between prescriptions and MS over the time windows (0–2, 2–5, and 5–10 years), and Poisson regression, the optimal approach to identify the length of diagnostic windows,^35,36^ which enabled specific points to be identified where there was a significant change in prescription rates before diagnosis of MS.

There are several limitations. First, in the current study, the authors could ascertain that a medication was prescribed, but not whether it was collected or used. Second, the study timeframe included the COVID-19 pandemic, potentially affecting prescription trends.^39^ However, in the current dataset, only 6% (*n* = 579/9662) of those in the case group were diagnosed from 2020 onwards. The authors therefore consider any impact on the results to be minimal. Third, this study did not use Hospital Episode Statistics to define case patients (limited completeness), but applied strict criteria to reduce diagnostic uncertainty in primary care. Fourth, this study used only 50 bootstrap iterations, which may introduce some variability in the estimated CIs. Increasing iterations could improve stability but would be computationally intensive for the large sample in this study. Because CIs were calculated using a normal approximation, the lower and upper bounds occasionally fell below zero or exceeded the study timeframe. In this study, the approach used by Price *et al* was applied to remain consistent with established methodology.^36^ Fifth, two cohorts were used: one requiring ≥5 years of registration and the other ≥10 years, each of which has inherent weaknesses. The 5-year cohort provides a larger sample but lacks complete long-term histories, whereas the 10-year cohort ensures longer look-back time but is smaller and potentially less generalisable. Another limitation concerns medication selection. Some medications, such as amitriptyline in the migraine category, are used for a potential symptom of MS but are also commonly prescribed around the time of MS diagnosis for neuropathic pain. This overlap may lead to misinterpretation, as some prescriptions might reflect treatment for emerging typical MS symptoms rather than true prediagnostic use that this study focused on. Finally, a convenience sample from a previous study was used — case patients and control patients were not matched on sex/general practice; however, sex was adjusted for in all analyses.

### Comparison with existing literature

Studies in the UK using CPRD data^18^ and in British Columbia, Canada^40^ found that individuals with MS were around two times more likely to have psychiatric morbidity and anxiety/depression symptoms in the 5–10 years before diagnosis. The current study supports these findings and suggests a clear diagnostic window, with anxiety/depression prescriptions rising 9–12 months before diagnosis in the all-prescription analysis. However, a potential 6–12 months recording lag in MS diagnosis must be considered,^41^ as some patients may already experience typical MS symptoms and be undergoing diagnostic work-up but not have a definitive diagnosis. Therefore, in some patients, an anxiety/depression prescription may indicate the onset of treatment when patients have already been referred and/or a provisional diagnosis rather than the prodrome.

This study found a positive link between MS diagnosis and ED prescription, with rates in the all-prescription analysis among the case group increasing above baseline at 60–63 months prediagnosis. The results were consistent with a European study, which linked sexual dysfunction within 5 years prediagnosis to increased subsequent MS risk (OR 1.47, 95% CI = 1.11 to 1.95),^20^ but differed from a CPRD study by Disanto *et al*, who found no association (*P*>0.1).^18^ The difference may be because of variations in study design. Disanto *et al* used symptoms, whereas the current study used prescriptions, which are of higher quality and completeness,^42^ and may also indicate more severe presentations requiring treatment.

A series of studies from Canada reported higher 5 years prediagnostic occurrences of gastritis, duodenitis,^43^ conditions of urinary system, including LUTS and UTI^17,44^, headache,^44,45^ constipation-related prescriptions, and antinauseants^43^ in patients with MS, which are consistent with the current study’s findings. The current study further showed that all prescription rates of ED, LUTS, and GI might increase above baseline rates from 5–6 years before MS diagnosis, supporting the idea that MS-related damage in the transmission of nerve signals controlling digestive function, bladder function, and sexual arousal in the central nervous system may start years earlier.^46–48^

Although in the all-prescription analysis, migraine and UTI prescriptions increased over time, the rates of increase slowed after the inflection points. This may be because of the relapsing–remitting nature of the MS prodrome, leading some patients to discontinue medications as symptoms subsided.

The current findings are consistent with UK and Canadian studies showing a positive relationship between sleep disorder-related symptoms^14,18^ in the 5 years prediagnosis and subsequent MS diagnosis. However, no clear inflection point was identified for this category in the all-prescription analysis, suggesting that sleep disorder prescriptions are more common in patients with MS, but no diagnostic window related to them could be defined for MS. This may be because of the gradual onset of symptoms over an extended timeframe in MS or the relapsing–remitting nature of the condition.

Despite a large sample in this study and evidence of increasing ORs in conditional logistic regression analyses closer to diagnosis, the CIs around the majority of inflection points were broad. By contrast, studies in cancer, where the current study’s authors and others have frequently employed these methods,^10,35,37^ often have sharply defined inflection points relating to prediagnostic activity. This may reflect the more gradual and relapsing–remitting nature of MS over a long timeframe accompanied by a more gradual increase in the prescribing rate.

### Implications for research and practice

Existing studies indicate that early treatment leads to better MS disease outcomes, and early diagnosis is a critical prerequisite for starting disease-modifying treatment in a timely way.^41^ The longer diagnostic delay in MS is related to greater periventricular brain lesions and higher risk of disability, both of which may ultimately reduce patients' quality of life.^7,41^ In the UK, the National Institute for Health and Care Excellence (NICE) guidelines recommend that GPs refer patients with symptoms or signs indicating possible MS to a neurologist for assessment.^49^ The latest NICE guidelines for MS diagnosis do not include symptoms of anxiety/depression, LUTS**,** UTI, migraine, GI, or ED as suspected symptoms of MS.^49^ This study indicates that prescribing for these symptoms and conditions is associated with MS and that prescription rates change months to years prediagnosis, which may signal undiagnosed/prodromal MS and indicate an opportunity for earlier diagnosis. These findings may help identify a high-risk population that may benefit from further targeted diagnostic investigations/monitoring to achieve earlier MS detection. However, prescriptions for these conditions are common in primary care and further research is needed to examine their predictive value alone and in combination with other features to determine whether they could add to current diagnostic tools or be incorporated into MS diagnostic prediction models^50,51^ in the future.

## Data

The data used in this study can be accessed from CPRD (https://cprd.com), with permission obtainable through Independent Scientific Advisory Committee.
